# The potential impact of a probiotic: *Akkermansia muciniphila* in the regulation of blood pressure—the current facts and evidence

**DOI:** 10.1186/s12967-022-03631-0

**Published:** 2022-09-24

**Authors:** Arun Prasath Lakshmanan, Selvasankar Murugesan, Souhaila Al Khodor, Annalisa Terranegra

**Affiliations:** 1 Precision Nutrition, Sidra Medicine, 26999, Doha, Qatar; 2 Laboratory of Microbiome and Biomarkers Discovery, Sidra Medicine, 26999, Doha, Qatar

**Keywords:** *Akkermansia muciniphila*, Hypertension, Gut microbiome, Metagenomic, Blood pressure, Gut microbiota

## Abstract

*Akkermansia muciniphila* (*A. muciniphila*) is present in the human gut microbiota from infancy and gradually increases in adulthood. The potential impact of the abundance of *A. muciniphila* has been studied in major cardiovascular diseases including elevated blood pressure or hypertension (HTN). HTN is a major factor in premature death worldwide, and approximately 1.28 billion adults aged 30–79 years have hypertension. *A. muciniphila* is being considered a next-generation probiotic and though numerous studies had highlighted the positive role of *A. muciniphila* in lowering/controlling the HTN, however, few studies had highlighted the negative impact of increased abundance of *A. muciniphila* in the management of HTN. Thus, in the review, we aimed to discuss the current facts, evidence, and controversy about the role of A*. muciniphila* in the pathophysiology of HTN and its potential effect on HTN management/regulation, which could be beneficial in identifying the drug target for the management of HTN.

## Introduction

*Akkermansia* spp., belong to the Verrucomicrobia family, with only two species identified so far, namely *Akkermansia muciniphila (A. muciniphila) *and *Akkermansia glycaniphila* [1, 2]. Both species are considered intestinal mucin-degrading bacterium; the former was initially isolated from the fecal samples of healthy Caucasian females in 2004, whereas the latter was isolated from fecal samples of reticulated python, *Malayopython reticulatus* in 2016 [1, 2]. *A. muciniphila* is an oval-shaped, non-motile, and gram-negative anaerobic bacteria, that grows well with an optimum temperature of 37 °C and pH of 6.5 [1], and it is present in wild animals, mice, hamsters, and humans [3]. Caputo et al*.* successfully sequenced *A. muciniphila*, directly from human stool samples using the whole-genome assembly method [4], and the abundance level of *A. muciniphila* in the human feces sample is approximately 3–4%. In rare conditions, the abundance level can increase up to 5% [5]. *A. muciniphila* is considered a potential probiotic due to its nature that can effectively use the gastrointestinal tract (GI) mucin [6] and possesses a unique survival mechanism, that is, the degradation of gastrointestinal mucin from the host causing the release of carbon and nitrogen sources for its survival [1, 7]. Also, its abundance level is modulated by dietary patterns and other changes in the mucin level [8]. In addition, it promotes the growth of other bacteria through a cross-feeding mechanism, mainly releasing amino acids and sugars during the GI mucin degradation process [8]. Its role has been studied in major diseases, such as diabetes mellitus, obesity, cardiovascular diseases, immune disorders, pregnancy complications, cancer, tumor, brain disorders, liver diseases, and kidney diseases. Its abundance level is very critical for normal physiological functions and any abnormality in its level is closely associated with the pathophysiology of these diseases [9–13]. Moreover, the drugs that we use to treat these diseases also impact *A. muciniphila*’s abundance level, mainly metformin [14], gemcitabine [15], paclitaxel [16], anti-PDI therapy [17], and some phytochemicals, such as andrographolide [18], puerarin [19], Bofutsushosan or Kampo [20] and resveratrol [21]. Additionally, *A. muciniphila* augments the drugs’ beneficial effects, mainly cisplatin—an anticancer drug- in lung cancer mice [21] and anti-PDI therapy through the production of CD4^+^T cells [17], metformin for glucose tolerance and glucose metabolism via secretion of glucose-regulating peptides and short-chain fatty acids (SCFAs) production in mice and humans [14, 22–24]. Thus, its interaction with the drugs is bidirectional. Interestingly, few recent studies from preclinical and clinical settings have shown that gut microbial dysbiosis can cause hypertension (HTN) [25, 26]. A Coronary Artery Risk Development in Young Adults (CARDIA) study conducted by Sun et al*.* found that both systolic and diastolic blood pressure (BP) is negatively correlated with the abundance of *A. muciniphila* [27]. Though it has been widely believed that *A. muciniphila* could play an unprecedented role in controlling the HTN, its role in the control of BP remains sparse. The current literature review suggests that there is no mechanistic evidence that unearths the direct role of *A. muciniphila* on the regulation of BP. Still, there is available indirect evidence that makes *A. muciniphila* a strong potential probiotic candidate for the control of BP. However, there are few contrary reports which highlight the negative role of *A. muciniphila* in controlling BP, especially in conditions where a higher inflammatory response is observed. Therefore, in this review, we aim to address and discuss the current knowledge on the potential roles of *A. muciniphila* in controlling/managing HTN.

## Mechanisms of gut microbiota-mediated HTN

So far, scientists worldwide have collected enormous data and made substantial progress in understanding the pathophysiology of HTN. Homeostasis of BP is regulated by numerous factors, such as the sympathetic nervous system through the release of vasoactive peptides, mainly dopamine, serotonin, and norepinephrine [25, 28, 29], renin–angiotensin–aldosterone (RAA) system [30], chronic kidney diseases (CKDs) [31], genetics [32], and various environmental and lifestyle factors [33, 34]. Recently the gut microbiota has been included in this long list, and it changed the paradigm of understanding the etiology of HTN [35, 36]. Scientists are now considering the microbiome as a central regulator of human health [37]. Gut microbiota dysbiosis is defined as the imbalance in the gut microbial composition that is linked to the disease state [38] can lead to HTN through the modulation of several factors, including short-chain fatty acids (SCFA)-mediated role in the RAAS system and immune system, sympathetic nervous system, trimethylamine-N-oxide (TMAO) pathway, nitric oxide pathway, energy homeostasis pathway, chronic inflammation pathway, microbiota-derived hydrogen-sulfide pathway, bile-acids, and uremic toxin pathway [39, 40]. Thus, gut microbiota plays a crucial role through a variety of checkpoints for the regulation of HTN. Humans contain trillions of microbiota, and each microbe in a group or alone plays a specific role in human physiology, especially probiotics such as genera *Lactobacillus* and *Bifidobacterium* [41]. Based on recent results from various animal and human studies, a new microbe—*A. muciniphila* has been included in the list of probiotics [6, 41, 42], which controls many metabolites, especially SCFA, lipopolysaccharide, TMAO, and hydrogen sulfide [43].

### Role of *A. muciniphila* in SCFAs regulated HTN

The SCFAs are produced from the diet mainly via saccharolytic fermentation of undigested carbohydrates, by anaerobic gut bacteria [43], and highly glycosylated mucin-degrading microbes, including *A. muciniphila* [5]. Humans produce approximately 500–600 mmol of SCFAs every day. Still, it strictly depends upon the amount of diet, especially the macronutrient content, such as carbohydrates, fibers, protein, and fat, that we consume [44]. The primary role of *A. muciniphila* is to degrade the mucin in the intestinal epithelial cells and supply energy sources (carbon and nitrogen) to goblet cells to the secretion of mucin again. This cycle should be in homeostasis [43]. In the secreted mucin, SCFAs such as acetate, propionate, butyrate, formate, isobutyrate, valerate, and isovalerate are produced in different concentrations, with acetate, propionate, and butyrate being the most abundant [45]. *A. muciniphila* mainly secretes acetate and propionate, which strengthen the gut barrier integrity through the regulation of tight junction proteins, such as occludin, claudins, and zona occludens [46]. Furthermore, the various clinical and preclinical studies using the 16s rRNA sequencing data have suggested that a decrease in the abundance of *A. muciniphila* could lead to the development of HTN [25, 28, 47]. Thus, all these results are based on association or correlation analysis. Still, to date, no direct studies (in-vivo, in-vitro, and clinical) use *A. muciniphila* to control BP. One of the major mechanisms of *A. muciniphila* in controlling BP could be the release of SCFAs from mucin [1, 7]. In the early days, SCFAs were thought to regulate BP through G-protein coupled receptors (GPR41 or FFAR3 and GPR43 or FFAR2) in humans, and Olfr78 in mice. In addition to this, presently, SCFAs regulate BP through GPR109a (also called HCA2 or NIACR1 or PUMA-G or HM47a) and OR51E2 in humans. In humans and mice, all three dominant SCFAs—propionate, acetate, butyrate, and subdominant SCFA—lactate regulate BP through these receptors, with propionate being the strongest ligand [48–53] to influence BP in humans via OR51E2.

Additionally, β-ionone—a volatile compound present in fruits, vegetables, and plants and cleaved from beta-carotene by an enzyme called carotenoid cleavage dioxygenase [54] influences BP in humans via OR51E2 [55, 56], but not through Olfr78 in mice [57]. The SCFAs – acetate and propionate [58–60], bind with the GPR41/GPR43 receptors, and promote the secretion of GLP-1/GLP-2 in the enteroendocrine L-cells of ileum and large intestine [1, 61]. In different diabetic and non-diabetic animal models, activation of GLP-1R (both exogenous and endogenous) has been demonstrated to lower BP by reducing sympathetic activity in the carotid body [62, 63]. Also, propionate involves in the release of a fat hormone–leptin, which alters the normotensive to hypertensive conditions. Zhao et al. have found that supplementation of *A. muciniphila* in chow diet-fed mice, reduced chronic low-grade inflammation by reducing plasma leptin and LPS [64], indicating that *A. muciniphila* might be playing more complex roles than what we knew so far. Besides, previous studies using *GPR41*^*−*^*/*^*−*^ mice, have demonstrated that the acetate and propionate-promoted release of leptin is mediated with the activation of the GPR43 receptor only, not through the GPR41 receptor [65, 66]. These data suggest that *A. muciniphila* supplementation might have profound effects on BP regulation through the GPR41 receptor rather than the GPR43 receptor, but this hypothesis needs to be further studied.

### Lipopolysaccharides synthesis

Gut barrier integrity plays an important role in preventing the leaking of synthesized lipopolysaccharides (LPS) and inflammatory markers into circulation, thereby regulating BP [67]. LPS is a group of lipopolysaccharide chemical complexes produced by the bacteria, especially from the outer membrane of the gram-negative bacteria, and it acts as a permeability barrier for the bacteria [68]. Also, LPS is a pathogen-associated molecular pattern (PAMP) molecule known to stimulate the Toll-Like Receptors (TLRs)—a class of pattern recognition receptors that potentiate the innate immune response. In particular, the TLR4 induces myddosome, which consists of adaptor proteins, such as MyD88, TIRAP, and serine-threonine kinases from the IRAK family, which receive the signals from TLR4, leading to the activation of inflammatory gene expression through NF-kB and AP-1 activation [69–71]. Disruption in the gut barrier integrity is closely associated with HTN due to increased intestinal inflammation and permeability [67, 72]. Kim et al*.* have demonstrated that hypertensive subjects had increased plasma LPS, intestinal fatty acid-binding protein (I-FABP), proinflammatory Th17 cells, and zonulin levels [72]. Supplementation of *A. muciniphila* improves the gut epithelial barrier integrity possibly through the reduction of circulatory LPS, inflammatory markers such as TNF-α, IL-6, CD36, SR-A1), IL-2, IFN-γ, IL-12p40, and MCP-1, and potentiating the expression of tight junction proteins (occludin, zonal occludens, and claudin-3, claudin-5) [73–76]. In addition, Chelakkot et al*.* have shown that *Akkermansia*-derived extracellular vesicles–Amuc_1100 reduce circulatory LPS levels [76] and activation of the TLR2 pathway [77], thereby promoting an anti-inflammatory effect. On the contrary, few studies have reported a higher level of *A. muciniphila* during disease progression [78, 79]. In our previous two studies, we found that a higher abundance of *A. muciniphila* was linked with the activation of LPS in CKD mice than in the control mice [80], and insufficient glycemic control in pediatric subjects with T1DM is linked to the higher level of *A. muciniphila* [81]. Thus, the level of *A. muciniphila* is critical for the pathophysiology of certain diseases where there is activation of an inflammatory-mediated pathway. Increased abundance of *A. muciniphila* degrades more mucus in the gut which damages the mucosal barrier, leading to the leak of inflammatory markers, LPS, and the activation of an inflammatory response [82]. Ganesh et al*.* have reported that *A. muciniphila* could differentially affect the gut ecosystem in relevance to the level of inflammation. In brief, they showed the presence of *A. muciniphila* in *Salmonella enterica* Typhimurium-infected gnotobiotic mice exacerbates the gut inflammatory response by increasing the mRNA levels of IFN-γ, IP-10, TNF-α, IL-2, IL-17, and IL-6 in the cecal and colonic tissue compared to the gnotobiotic controls mice [83], which explains *A. muciniphila’s* ability to disturb the host mucus homeostasis through the excessive mucin degradation process and eventually disturbs the gut ecosystem. Li et al*.* have demonstrated that the supplementation of *A. muciniphila* prevents the severity of atherosclerotic lesions by reducing metabolic endotoxemia [10]. To support this hypothesis, a recent study by Dan et al*.* on the Chinese population has demonstrated that the abundance of *A. muciniphila* is greatly elevated in hypertensive subjects [84]. Also, Cekanaviciute et al*.* have reported that *A. muciniphila* promotes Th1 lymphocyte differentiation [85]. Further, a study on the Brazilian population conducted by Silveira-Nunes et al*.* found a higher abundance of *Akkermansia* along with the increased TNF-α/IFN-γ ratio in the hypertensive subjects [86], suggesting the differential role of *Akkermansia* in mediating HTN.

So, activation of the LPS pathway might be detrimental in inciting hypertension through various mechanisms, such as endothelial dysfunction through the release of inducible nitric oxide, activating TLR4, and vasculature inflammation through the release of NADPH oxidase-dependent free radicals [87–89]. On the other hand, *Akkermansia* supplementation reduced low-grade inflammation by suppressing circulatory LPS in HFD-diet mice [64].

### Trimethylamine-N-oxide

Several studies demonstrated that trimethylamine-N-oxide (TMAO) is a pathogenic metabolite formed by the gut microbiota and act as an independent risk factor for cardiovascular diseases, including HTN [90–93]. TMAO is formed by the oxidation of the intermediate metabolite TMA from the dietary L-carnitine, phosphatidylcholine, lecithin, and γ-butyrobetaine (γ-BB) via flavin monooxygenase 3 (FMO3) enzyme from gut microbes [94]. TMAO is involved in the development of hypertension through the elevation of vascular oxidative stress and endothelial dysfunction [95]. Xu et al. reported that the supplementation of *A. muciniphila* reverses the high expression of FMO3 in obesity conditions [76]. Also, recently Zhou et al*.* studied the relationship between circulating plasma TMAO levels and HTN. They found that TMAO levels are higher in patients with HTN than those without HTN [96]. Hsu et al*.* reported that a higher TMA level is associated with high BP load and abnormal 24-h ambulatory BP monitoring profile, along with the reduced abundance of genus *Akkermansia* [92]. Plovier et al*.* demonstrated that the supplementation of *A. muciniphila* significantly decreased plasma levels of TMAO and TMA and a two-fold reduction of FMO3 expression in mice [97]. Luo et al. demonstrated that cold exposure modulated the *A. muciniphila* abundance level in rats, thereby causing excessive secretion of TMAO which elevated the susceptibility to atrial fibrillation (AF) condition mainly through the enhanced infiltration of M1 macrophages in atria and increased protein expression of Casp1-p20 and cleaved GSDMD. The oral supplementation of *A. muciniphila* in rats significantly ameliorated the pro-AF property induced by cold exposure [98]. These results indicate a positive correlation between a higher level of TMAO or TMA with the HTN and a negative association with the abundance of the genus *Akkermansia*.

### Hydrogen sulfide

Another potential mechanism by which the *A. muciniphila* regulates BP could be the release of endogenous hydrogen sulfide (H_2_S), as it is involved in the sulfate reduction process [99]. Rosario et al*.* have demonstrated that *A. muciniphila* produced H_2_S together with *Escherichia* spp., which has been considered a potential regulator of vascular homeostasis, possibly through the regulation of vascular tone and inflammation, antioxidant mechanism, vascular cell proliferation, and apoptosis [100, 101]. Several studies have pointed out that the H_2_S levels are inversely associated with hypertensive disease severity [102–104]. Supplementation of H_2_S reduces BP in experimental animal models [105, 106]. On the contrary, H_2_S has been implied in activating proinflammatory response [107, 108], and its higher level could cause inflammation in the gut [99]. Interestingly, *A. muciniphila* has been proposed to use the H_2_S for the production of cysteine [43], and cysteine is well-known for its anti-hypertensive effect [109], thereby it could control BP regulation. In addition, when there is an activation of the inflammatory response, the relation between *A. muciniphila* and H_2_S might turn on pathologic response rather than the protective response, because both entities have been found to have a differential role. There is a possibility that a high level of *A. muciniphila* can produce a high amount of H_2_S, which might elevate the BP due to the activation of an inflammatory response [99]. This is a mere hypothesis and must be proved experimentally, but the evidence strongly encourages this concept. Recently, pangenomic analysis of *A. muciniphila* revealed that the phylotype AmI contains genes required for the assimilatory sulfate reduction (ASR) process and thereby possibly increasing H_2_S level.

### Role of *A. muciniphila* on risk factors that cause HTN

#### Chronic kidney disease (CKD)

CKD is closely associated with the pathogenesis of HTN, and the basic mechanisms behind the development of CKD-associated HTN are salt retention, endothelial dysfunction, sympathetic overactivity, volume overload, and abnormal hormonal level [110]. A classic manifestation of CKD is chronic inflammation, which causes a leaky gut, results in translocation of endotoxin, bacterial fragments, and uremic toxins in the circulation, and eventually leads to the accumulation of gut microbiota-derived uremic toxins in the circulation [111]. Gut microbiota-derived uremic toxins are mainly indoles (indole-3-acetic acid, indoxyl glucuronide, indoxyl sulfate (IS), kynurenine, kynurenic acid, melatonin, and quinolinic acid), phenols (hydroquinone, p-cresyl glucuronide, p-cresyl sulfate (PCS), phenol, and phenylacetic acid) [112]. The general effects of these uremic toxins are vascular dysfunction, such as increased vasoconstriction and decreased vasorelaxation, decrease in NO production, induction of oxidative stress, promotion of vascular cell apoptosis, and vascular remodeling [113]. Also, IS and PCS have been reported to promote vascular calcification [114]. Collectively all these detrimental effects are significantly associated with the pathogenesis of HTN. The role of *A. muciniphila* in gut-microbiota-derived toxins mediated vascular dysfunction leading to the HTN is controversial because of its differential role and abundance level in renal HTN. Li et al*.* have reported *A. muciniphila* level is reduced in association with elevated proinflammatory cytokines, such as IL-6, IL-8, and IFN-γ, and decreased anti-inflammatory cytokines, such as IL-4 and IL-10 in the CKD subjects in comparison with the HC subjects [79]. But, several studies have shown that significant elevation in the abundance of *A. muciniphila* level was observed in CKD animal models and human subjects [12, 80, 115, 116] along with the increase of IS and PCS [80]. So, the precise role of *A. muciniphila* in the context of renal HTN caused by CKD is still unclear, but it seems that *A. muciniphila* might augment the development of renal HTN rather than prevent it because *A. muciniphila* promotes inflammation, and in renal HTN a higher level of the inflammatory response has been reported [117] which might potentiate the level of *A. muciniphila*.

#### Renin-angiotensin system

The role and the activation of the renin-angiotensin (RAS) pathway in HTN has been extensively studied [118] and there is a possibility that a greater connection between *A. muciniphila* and the RAS pathway will be unveiled by the following studies: Buford et al*.* have reported that oral administration of the probiotic *Lactobacillus paracasei* increased the expression of the Angiotensin (1-7), an Angiotensin-I vasopeptide, and significantly enriched the abundance of *A. muciniphila* [119]. Roshanravan et al*.* also have concluded that the protective effects of sodium butyrate and inulin supplementation on type 2 diabetes mellitus can act via the enrichment of *A. muciniphila* on the angiotensin signaling pathway [120]. Duan et al*.* pointed out that the level of *A. muciniphila* was less in the ACE2^−/y^ mice than in the ACE^−/y^-Akita mice and it showed a reduced number of myeloid angiogenic cells (MACs) without a significant increase in inflammatory monocytes. Furthermore, the administration (exogenous) of MACs restored gut barrier integrity and altered the gut microbiota [121]. Recently, a study conducted by Suzuki et al*.* showed that the altitude variation approximately ranging from 33 to 4000 m from the sea level, greatly affects the composition of gut microbiota in wild-type mice [122]. They found that the relative abundance of genus *A. muciniphila* was the most negatively correlated with the increasing altitude [122].

Furthermore, their predicted functional metagenome analysis indicated that the KEGG pathway—RAS was positively correlated with the increasing altitude, suggesting a strong association between *A. muciniphila* and the RAS pathway [122]. In addition, Robles-Vera et al*.* demonstrated that 5 weeks of treatment of renin-angiotensin-II blocker—losartan on spontaneously hypertensive rats (SHR) significantly reduced the gut dysbiosis and sympathetic drive in the gut, which possibly can contribute to the reduction of BP by modulating the immune system in the gut [123]. Also, they reported that losartan treatment significantly restored the abundance level of genus *Akkermansia* along with the other genera, such as *Pedobacter* and *Lactobacillus* in SHR [123]. Thus, these studies have pointed out the potential link between RAS and *A. muciniphila*, and establishing these two entities warrants further mechanistic studies.

#### Endothelial dysfunction/endothelial-derived nitric oxide pathway

Endothelial dysfunction is a type of non-obstructive chronic artery disease (CAD) in which blood vessel constricts instead to relax, leading to chronic chest pain [124]. The molecular mechanism for the occurrence of endothelial dysfunction is an imbalance between endothelial-derived relaxing factors (endothelin-1, angiotensin-II, thromboxane A_2_, and reactive oxygen species), and endothelium-derived hyperpolarizing factors (NO and prostacyclin) [125, 126]. Among them, endothelium-derived NO is considered as a potent vasodilator, and its implication in the regulation of BP through various mechanisms, such as inhibition of vasoconstriction, platelet aggregation, angiogenesis, oxidative stress, inflammation, and atherogenesis, and its reduced level would have opposite effects on vascular smooth muscle cells are well-established [127, 128]. Gut microbiota is closely linked to vascular dysfunction regulation [129]. Wang et al. reported that berberine improves vascular dysfunction via the modulation of gut microbiota possibly through the inhibition of TMAO production in angiotensin II-induced hypertensive mice [130]. Oral supplementation of *A. muciniphila* in Apolipoprotein E knockout mice (ApoE^−/−^) model to study the endothelial dysfunction along with atherogenesis, has shown the reduction in the metabolic endotoxemia-induced inflammatory chemokines, such as ICAM-1, MCP-1, and TNF-α on the endothelium [10]. Also, inulin-type fructans supplementation improved the endothelial dysfunction in ApoE^−/−^ through the eNOS-NO pathway, along with the increment in the abundance of *A. muciniphila* [131]. Lee et al*.* have found a significant correlation between vascular dysfunction and the abundance of *A. muciniphila* levels, and dapagliflozin, an antidiabetic drug, improves the vascular dysfunction along with the increase of *A. muciniphila* level in the T2DM [132]. Haywood et al*.* have demonstrated that endothelial dysfunction is reversed with the overexpression of endothelial cell insulin-like growth factor 1 receptor (ECIGF-1R), along with the increase of *A. muciniphila* abundance in obesity conditions [133]. Thus, it is evident that *A. muciniphila* has a role in endothelial function through NO release and inhibition of proinflammatory chemokines, and the improvement in vascular/endothelial dysfunction might restore elevated BP. Surprisingly, Neyrinck et al*.* have reported that endothelial dysfunction was improved with the reduction of *A. muciniphila* abundance in ApoE^−/−^ mice and suggested the reason for the negative role of *A. muciniphila* could be due to an individual’s health and microbiota pattern [134].

#### Epigenetic mechanisms

The epigenetic mechanisms are a set of post-translational modifications that can regulate the gene expression transiently without affecting the DNA sequence and involve chemical modification of DNA. Still, the changes are reversible in influencing the phenotype [135]. It results from an interplay between DNA and environmental factors [136]. The most common epigenetic mediators are DNA methylation and histone modifications, and recently long-coding RNA has been identified as another epigenetic mediator [136]. Richard et al*.* have found that methylation at cg08035323 influenced BP, and BP influenced methylation at different sites, such as cg00533891, cg00574958, and cg02711608 [137]. Also, DNA methylation and histone modifications (methylation and acetylation) with various drug treatments (valproic acid, decitabine, and 5-aza-2ʹ-deoxycytidine) have been associated with BP reduction in animal models [138–141]. Cardinale et al*.* have reported that histone deacetylase (HDAC) inhibition attenuates hypertension in Spontaneous Hypertensive Rats (SHR) [140], whereas *A. muciniphila* was found to potentiate HDAC3 and HDAC5 in improving the host lipid metabolism condition [61]. Furthermore, Jabs et al*.* have identified that *A. muciniphila* greatly affects the N6-methyladenosine modifications in mRNA in mono-associated mice [142]. Thus, the clarity on epigenetic mechanisms by *A. muciniphila* requires further studies as we have minimal available data.

### The DASH diet, Mediterranean diet, and weight-loss intervention program on *A. muciniphila* to control BP

Diet is an essential modulator of gut microbiota [143–145], and the specific dietary pattern called the dietary approach to stop hypertension (DASH) has been proposed to control or lower BP [146]. The DASH diet promotes a diet that is adjusted to be lower in sodium and rich in potassium, magnesium, and calcium nutrients [146], which causes a reduction in high BP [147]. Maifeld et al*.* demonstrated that fasting followed by the modified DASH diet reduced the BP in patients with metabolic syndrome (MetS) through an altered gut microbiome, especially increasing the abundance of *A. muciniphila*, which in turn, increases SCFAs either locally or systemically [148]. Also, beta-glucan supplementation for four weeks in patients with MetS had a higher outcome in patients with a higher baseline *A. muciniphila* abundance [149]. Another weight-loss intervention study in patients with MetS had improved BP by enhancing the abundance of *A. muciniphila and F. parusnitzii* [150]. American Heart Association (AHA) recommends the Mediterranean diet (MD) as a healthy eating style; it improves both systolic and diastolic BP, and arterial stiffness [151–154]. Also, MD has been reported to increase the relative abundance of *A. muciniphila* in the stool samples from participants with different body weight compositions [155]. Thus, *A. muciniphila* could be a potential entity in lowering the BP in patients with MetS.

### Interaction between anti-hypertensive drugs and *A. muciniphila*

Most hypertensive drugs, such as ARB, ACE inhibitors, beta-blockers (BBs), and calcium channel blockers (CCBs), are administered orally. After the administration by oral route, it is metabolized into pharmacologically active metabolites or toxic based on the molecule's structure [156]. These active molecules can be further metabolized by the rich enzyme repositories of the gut microbiome [157, 158]. The interaction between the drug and gut microbiome is bidirectional. Thus, identifying gut bacteria and their communities, which produce drug-metabolizing microbial proteins, can contribute to improving the drug’s effect and can be converted into new drugs and reduce the adverse effect. Understanding the relationship between the gut microbiome and HTN drug bioavailability can help us better understand individual variation, which can be useful in precision and personalized medicine. Unfortunately, the literature review indicates the need to study these two entities (anti-hypertensive drugs vs *A. muciniphila*) as we have very little evidence. For example, Li et al*.* reported that amlodipine aspartate and amlodipine besylate, a CCBs, improved the NFALD condition in mice with HTN through the enhancement of *A. muciniphila*, which potentiates the intestinal expression of antimicrobial peptide Reg3g [159]. As mentioned earlier, losartan treatment significantly improved the hypertensive condition in SHR rats possibly through the restoration of gut dysbiosis and modulation of immune response, along with the increased the abundance of *A. muciniphila* [123]. Furthermore, minocycline, an age-old antibiotic, is demonstrated to lower BP, and administration of minocycline in an SHR animal model decreased BP with an increase of *A. muciniphila* abundance [28]. Also, *A. muciniphila* secreted proteins induces the uptake of Ca^++^ through Ryanodine Receptors (RYr), a family of Calcium channel, in an enteroendocrine cell line and increase the synthesis of reactive oxygen species (ROS) [160]. Nonetheless, more research is needed to fully understand the possible bidirectional role of *A. muciniphila* and anti-hypertensive drugs.

## Can *A. muciniphila* be considered in the field of pharmacomicrobiomics?

Numerous studies have highlighted the presence of microbiota in the gut as they play various crucial roles in improving health, including lowering BP through complex mechanisms. Pharmacomicrobiomics describes the differential effect of the microbiome on the pharmacokinetic profile of the drugs [161] with a focus on the microbiota-drug interaction. *Akkermansia* could be one of the potential regulators in the field of pharmacomicrobiomics as they potentiate drugs’ effects, such as PD-1/PD-L1A blockers, metformin, rifaximin, resveratrol, vancomycin, and vitamin-D in variety of disease conditions. A cross-sectional study using atorvastatin in hypercholesterolemic patients focused on understanding the impact of the gut microbiota revealed that untreated patients had an increase in inflammatory microbes, such as *Collinsella*, and *Streptococcus,* and treated patients had an increase in anti-inflammatory microbes such as *A. muciniphila* and *F. parusnitzii* [162]. A study among Brazilian hypertensive participants revealed that *Akkermansia* was highly abundant in addition to *Lactobacillus* and *Eggerthella* than in the normotensive group. Thus, we can speculate that *A. muciniphila* may play a role in HTN, by promoting the proinflammatory environment in the host. Considering all these available data, it suggests that *A. muciniphila* can be a potential candidate to be considered in the field of pharmacomicrobiomics.

## Pangenome of *A. muciniphila* and its role in lowering BP

Pangenome is a set of entire genes in a collection of an organism's genomes [71]. It consists of a core genome shared by about 99% of the strains and a dispensable genome. Cluster analysis of microbial species genomes can offer insight into multiple fields, including taxonomy, evolutionary dynamics, strain diversity, strain identification, and reverse vaccinology [163, 164]. A comparative study to explore the population structure, genomic and functional diversity of *A. mucinphila* by comparing 39 genomes of its isolates showed that it contains 5644 unique proteins, and 106 new *A. muciniphila *metagenome-assembled genomes (MAGs) of human, pig, and mouse gut microbiomes. Focusing on the functional diversity analysis revealed that its pangenome consists of 198 CAZymes coding genes higher than other members of gut microbiota [165]. Becken et al. reported that human (obese) isolates of *A. muciniphila* have four distinct phylotypes (AmI to AmIV) and genotypic and phenotypic diversity [166]. These phylotypes of *A. muciniphila* have more affinity toward amino sugar and nucleotide sugar metabolism, fructose and mannose metabolism, and carbon metabolism pathways [166]. They reported that several phylogroup-specific phenotypes impacted colonization of the GI tract and modulated several host functions, such as oxygen tolerance, adherence to epithelial cells, and iron and sulfur metabolism. They also found that phylotype AmI was the most prominent in these populations, and this phylotype AmI contains more genes required for ASR, interestingly it was absent in the phylotypes AmII and AmIV. Due to a higher number of ASR genes present in the phylotype AmI, more sulfate is converted into H_2_S in the canonical ASR pathway, which increases the synthesis of sulfur-containing anti-hypertensive amino acids, such as cysteine and methionine. Thus, it shows that the pangenome of *A. muciniphila*, especially the phylotype AmI could be a potential target in lowering BP.

## Obstacles and future perspectives

Even though numerous studies have revealed the gut microbial composition and metabolic roles in various diseases, it is still unclear how this knowledge can be applied to clinical medicine. For a better understanding of the gut microbiota and its dynamic nature during the onset, progression, and response to the pharmacological treatment, the application of longitudinal and interventional studies is essential to descriptive studies which only show dysbiosis. To improve drug efficacy, the microbiome can be considered a vital factor to use in non-responsive and variable-responsive patients. Subsequently, standardized strategies for reprogramming the gut microbiota should be implemented. Furthermore, other secondary metabolites produced by the gut microbiota from xenobiotics and dietary molecules should be taken into account, which will also interact with drugs [167]. Furthermore, currently available, a limited number of public data (16s rDNA sequencing) from studies that involved both humans and small rodents did not provide conclusive evidence as it shows a moderate but not a significant decrease in the abundance of *A. muciniphila* in hypertensive conditions (Table 1 and 2), which highlight the need of more studies on *A. muciniphila* in hypertension. Also, the literature review indicates that there is enormous scope in elucidating the potential role of *A. muciniphila* on different pathways, such as the H_2_S pathway, RAS pathway, and epigenetic mechanisms. As currently, we have limited information on these potential regulatory pathways. Recently, Liu et al*.* reported that the isolation of miR-30d from feces of multiple sclerosis (MS) patients and its administration significantly improved the MS-like symptoms by expanding the abundance of *A. muciniphila* [168]. This fascinating finding opens up a new path in the field of microbiome research, and it has the potential to impact disease treatment in the near future enormously. Advanced multi-omics approaches, such as metabolomics, lipidomics, and genomics with microbiomics, as well as the integration of these data, can help to dissect the mechanisms through which the gut microbiota modulates the host-drug response [169].

**Table 1:** Current literature review about the role of *A. muciniphila* on small rodent HTN model

Study title	Animal	Comparison	*Akkermansia muciniphila* abundance	References
High-salt (HS) diet-induced hypertension model	Sprague–Dawley rat	Control vs HS groups	No change	Ding et al*.* [170]
Acute Intra-abdominal (aIAH) HTN model	Sprague–Dawley rat	Control vs IAH groups	No change	Leng et al*.* [171, 172]
Spontaneous-hypertensive rat (SHR) models	SHR rat	SHR vs WKY2 groups	Decreased [173] and increased (not statistically significant), no change	Abboud et al*.* [173], Singh et al*.* [174], Li et al*.* [170], Wu et al*.* [175, 176]
High carbohydrate and fat diet-induced hypertension	Wistar rats	diet-induced HTN vs control groups	Increased (not statistically significant)	Thomaz et al*.* [177]
High fat diet-induced cardiometabolic disorders	Wistar rats	HFD diet vs control groups	No change	de Araujo Henriques Ferreira et al*.* [178]
High fat-diet underwent vertical sleeve gastrectomy (VSG) surgery	C57BL/6 J	Sham vs VSG groups	No change	McGavigan et al*.* [179]
Altitude variation model	Mus musculus domesticus	Comparison at different altitude	Decreased in higher altitude	Suzuki et al*.* [122]

**Table 2:** Current literature review about the role of *A. muciniphila* on human HTN

Study title	Population	Comparison	*Akkermansia muciniphila* abundance	References
Predicted gut microbiomes from a multi-site blood pressure study	Australian	Normotensive vs hypertensive groups	No change	Nagai et al*.* [180]
Gut metagenomic signature in hypertension: a cross-sectional study	Española	Normotensive vs hypertensive groups	No change	Calderon-Perez et al*.* [181]
The human microbiome correlates with risk factors for cardiometabolic disease across an epidemiologic transition	African-origin	Normotensive vs hypertensive groups	Decreased in normotensive groups (but not statistically significant)	Fei et al*.* [182]
Fasting alters the gut microbiome with sustained blood pressure and body weight reduction in metabolic syndrome patients	Germans	Fasting + DASH diet vs DASH diet	Increased in Fasting + DASH groups	Maifeld et al*.* [148]
Hypertension microbial diversity	Chinese	Normotensive vs hypertensive groups	No change	Human University of Chinese Medicine [183]
Washed microbiota transplantation lowers blood pressure in patients with hypertension	Chinese	Normotensive vs hypertensive groups	No change	Zhong et al*.* [184]

## Conclusions

**Fig. 1:**
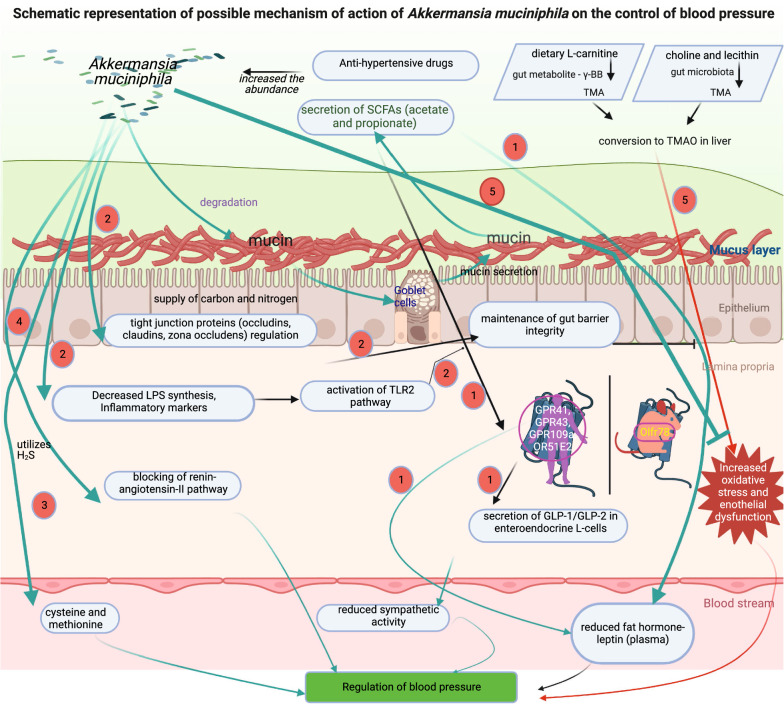
Schematic representation of the possible mechanism of action of *A. muciniphila* on the control of BP. The potential possible mechanisms of *A. muciniphila* to control the BP are (1) the degradation of mucin to secrete the SCFAs, especially acetate and propionate that reduces plasma leptin secretion and sympathetic activity through the secretion of GLP-1/GLP-2 in enteroendocrine L-cells via G-protein-coupled receptors; (2) maintenance of gut barrier integrity through the regulation of the tight junctions proteins (occludins, claudins, zona occludens), and activation of TLR2 pathway through the reduction of LPS synthesis and inflammatory markers; (3) utilization of H_2_S to produce cysteine which improves the BP; (4) direct action (possibly) on the renin-angiotensin-II pathway, and (5) reduction of oxidative stress induced by TMAO through the dietary L-carnitine metabolite

According to current evidence from clinical and preclinical studies, the gut microbiota plays an essential role in hypertension. *A. muciniphila*, in particular, is regarded as an important beneficial microbe because it regulates several molecular pathways, chemical entities, and dietary metabolites involved in BP regulation (Fig. 1). Our knowledge of these interactions and their effects on HTN is limited. Through well-controlled studies, researchers will better understand the molecular mechanisms by which the gut microbiota regulates HTN and the host response to drugs, could result in improved clinical outcomes and bring a step closer to precision medicine.

## Data Availability

Not applicable.

## Abbreviations

*A. muciniphila*: *Akkermansia muciniphila*
ACE: Angiotensin convertingangiotensin-converting angiotensin-converting enzyme
ACE2: Angiotensin-converting enzyme 2
AP-1: Activator protein 1
ApoE: Apolipoprotein E
ASR: Assimilatory sulfur reduction
BP: Blood pressure
CD36: Cluster of differentiation 36
CKD: Chronic kidney diseases
DASH: Dietary approach to stop hypertension
ECIGF-1R: Endothelial cell insulin-like growth factor 1 receptor
ECIGF-1R: Endothelial cell insulin-like growth factor 1 receptor
eNOS: endothelial nitric-oxide synthase
FFAR2: Free fatty acid receptor 2
FFAR3: Free fatty acid receptor 3
GI tract: Gastrointestinal tract
GLP1: Glucagon-like peptide-1
GLP2: Glucagon-like peptide-2
GPR: G-protein coupled receptor
GPR109a: G-protein coupled receptor 109a
GPR41: G-protein coupled receptor 41
GPR43: G-protein coupled receptor 43
H_2_S: Hydrogen sulfide
HC: Healthy control
HFD: High-fat diet
HTN: Hypertension
I-FABP: Intestinal fatty acid-binding protein
IFN-γ: Interferon-gamma
IL-2: Interleukin 2
IL-4: Interleukin 4
IL-6: Interleukin 6
IL-10: Interleukin 10
IP-10: Interferon γ-induced protein 10
IRAK: Interleukin-1 receptor-associated kinase
IS: Indoxyl sulfate
LPS: Lipopolysaccharide synthesis
MAG: Metagenome-assembled genomes
MCP-1: Monocyte chemoattractant protein-1
MD: Mediterranean diet
MetS: Metabolic syndrome
MyD88: Myeloid differentiation primary response 88
NFALD: Non-alcoholic fatty liver disease
NF-кB: Nuclear factor kappa-light-chain-enhancer of activated B cells
NO: Nitric oxide
Olfr78: Olfactory receptor 78
OR51E2: Olfactory receptor family 51 subfamily E member 2
PCS: P-cresyl sulfate
PD-1: Programmed cell death protein 1
PDL1A: Programmed cell death ligand 1A
RAA system: Renin–angiotensin–aldosterone system
RAS pathway: Renin-angiotensin system pathway
ROS: Reactive oxygen species
RYr: Ryanodin receptors
SCFAs: Short-chain fatty acids
SHR: Spontaneously hypertensive rats
SR-A1: Scavenging receptor A1
T1DM: Type 1 diabetes mellitus
T2DM: Type 2 diabetes mellitus
Th1: T-helper 1
Th17: T-helper 17
TIRAP: Toll/interleukin-1 receptor domain-containing adapter protein
TLR2: Toll-like receptor 2
TLR4: Toll-like receptor 4
TLRs: Toll-like receptors
TMA: Trimethylamine
TMAO: Trimethylamine-N-oxide
TNF-α: Tumor necrosis factor-alpha
